# Explicable prioritization of genetic variants by integration of rule-based and machine learning algorithms for diagnosis of rare Mendelian disorders

**DOI:** 10.1186/s40246-024-00595-8

**Published:** 2024-03-21

**Authors:** Ho Heon Kim, Dong-Wook Kim, Junwoo Woo, Kyoungyeul Lee

**Affiliations:** Research and Development Center, 3billion, 14th floor, 416 Teheran-ro, Gangnam-gu, Seoul, 06193 Republic of Korea

**Keywords:** Explainable AI, Clinical genome interpretation, Variant prioritization, Mendelian disorder

## Abstract

**Background:**

In the process of finding the causative variant of rare diseases, accurate assessment and prioritization of genetic variants is essential. Previous variant prioritization tools mainly depend on the in-silico prediction of the pathogenicity of variants, which results in low sensitivity and difficulty in interpreting the prioritization result. In this study, we propose an explainable algorithm for variant prioritization, named 3ASC, with higher sensitivity and ability to annotate evidence used for prioritization. 3ASC annotates each variant with the 28 criteria defined by the ACMG/AMP genome interpretation guidelines and features related to the clinical interpretation of the variants. The system can explain the result based on annotated evidence and feature contributions.

**Results:**

We trained various machine learning algorithms using in-house patient data. The performance of variant ranking was assessed using the recall rate of identifying causative variants in the top-ranked variants. The best practice model was a random forest classifier that showed top 1 recall of 85.6% and top 3 recall of 94.4%. The 3ASC annotates the ACMG/AMP criteria for each genetic variant of a patient so that clinical geneticists can interpret the result as in the CAGI6 SickKids challenge. In the challenge, 3ASC identified causal genes for 10 out of 14 patient cases, with evidence of decreased gene expression for 6 cases. Among them, two genes (*HDAC8* and *CASK*) had decreased gene expression profiles confirmed by transcriptome data.

**Conclusions:**

3ASC can prioritize genetic variants with higher sensitivity compared to previous methods by integrating various features related to clinical interpretation, including features related to false positive risk such as quality control and disease inheritance pattern. The system allows interpretation of each variant based on the ACMG/AMP criteria and feature contribution assessed using explainable AI techniques.

**Supplementary Information:**

The online version contains supplementary material available at 10.1186/s40246-024-00595-8.

## Introduction

Rare disorders (RDs), 80% of which have genetic causes, are estimated to affect approximately 6% of the global population [1]. The advent of next-generation sequencing (NGS) has had a profound impact on the human genetics/genomics and medical genetics fields by revolutionizing the way rare disease diagnostics and disease gene discovery are performed [2]. The length of the diagnostic odysseys of RD patients can be greatly shortened as genetic variants in all possible disease genes can be assessed simultaneously in an unbiased manner [3]. However, variant interpretation remains difficult, and many variants are still classified as having uncertain significance although numerous in-silico tools [4] are being developed to predict how damaging a variant could be. Rigorous assessments of variants require gathering the latest information from various public and private databases and assessing the variant’s pathogenicity according to the relevance of the patient’s symptoms to the reported phenotypes of each gene/disease [5]. Consequently, diagnosis of genetic disorders based on the genome of patients requires enormous time and effort from clinical geneticists. To address this issue, various tools have been developed to efficiently detect causal genes and variants by prioritization.

Variant prioritization based on genotype—phenotype knowledge along with variant data is typically used to find the causative variant(s) [2]. There are several tools, such as Exomiser [6], LIRICAL [7], and AMELIE [8], that use clinical phenotype data of patients according to Human Phenotype Ontology (HPO) [9] to prioritize each candidate variant based on accepted phenotypic knowledge.

Exomiser combines variant-based scores and gene-based scores to calculate a final score using a logistic regression model. Variant-based scores are determined based on variant frequency and pathogenicity prediction by MutationTaster [10], PolyPhen-2 [11], and SIFT [12]. Exomiser filters variants based on criteria such as variant type, allele frequency, variant call quality [13], and inheritance patterns. Although the variant filtering process helps improve variant prioritization accuracy by removing false positives, some diagnoses could be missed due to low quality in the variant call format (VCF), high allele frequencies, and incomplete penetrance [2].

LIRICAL uses a statistical framework to estimate the posterior probabilities of candidate diagnoses based on the likelihood ratio (LR). LIRICAL uses in-silico pathogenicity scores to calculate the LR of the observed genotype and then combines the result with the LR for the phenotypes to obtain the posttest probability of each disease for the given observations.

A unique trait of AMELIE is that it parses 29 million PubMed abstracts and hundreds of thousands of full-text articles to support the diagnosis. AMELIE uses 27 features extracted from 6 different information categories necessary for molecular diagnosis. It considers features related to inheritance mode, AVADA-extracted variants, patient phenotypes, and article and variant types, and finally, in-silico pathogenicity scores [14] and gene-level intolerance [15, 16]. A logistic regression classifier based on those features was trained using simulated patient data.

Previous models largely depend on in-silico pathogenicity scores and known pathogenic variants to assess patient genotypes. However, the pathogenicity of genetic variants needs to be assessed differently depending on the associated gene, disease, and family history according to the ACMG/AMP guidelines [17], which are the standard guidelines for diagnosing patients recommended by the American College of Medical Genetics and Genomics and the Association for Molecular Pathology. Although in-silico prediction can be useful in finding novel pathogenic variants, other contexts of the variant also need to be considered. For example, when in-silico prediction is used as supporting (PP) evidence of variant pathogenicity, the patient can be diagnosed by the variant only if very strong (PVS) or strong (PS) evidence of pathogenicity is also present. Variant prioritizations without further evidence are often either uninterpretable, or not precise enough to identify the causative variant in the first place [5].

**Fig. 1:**
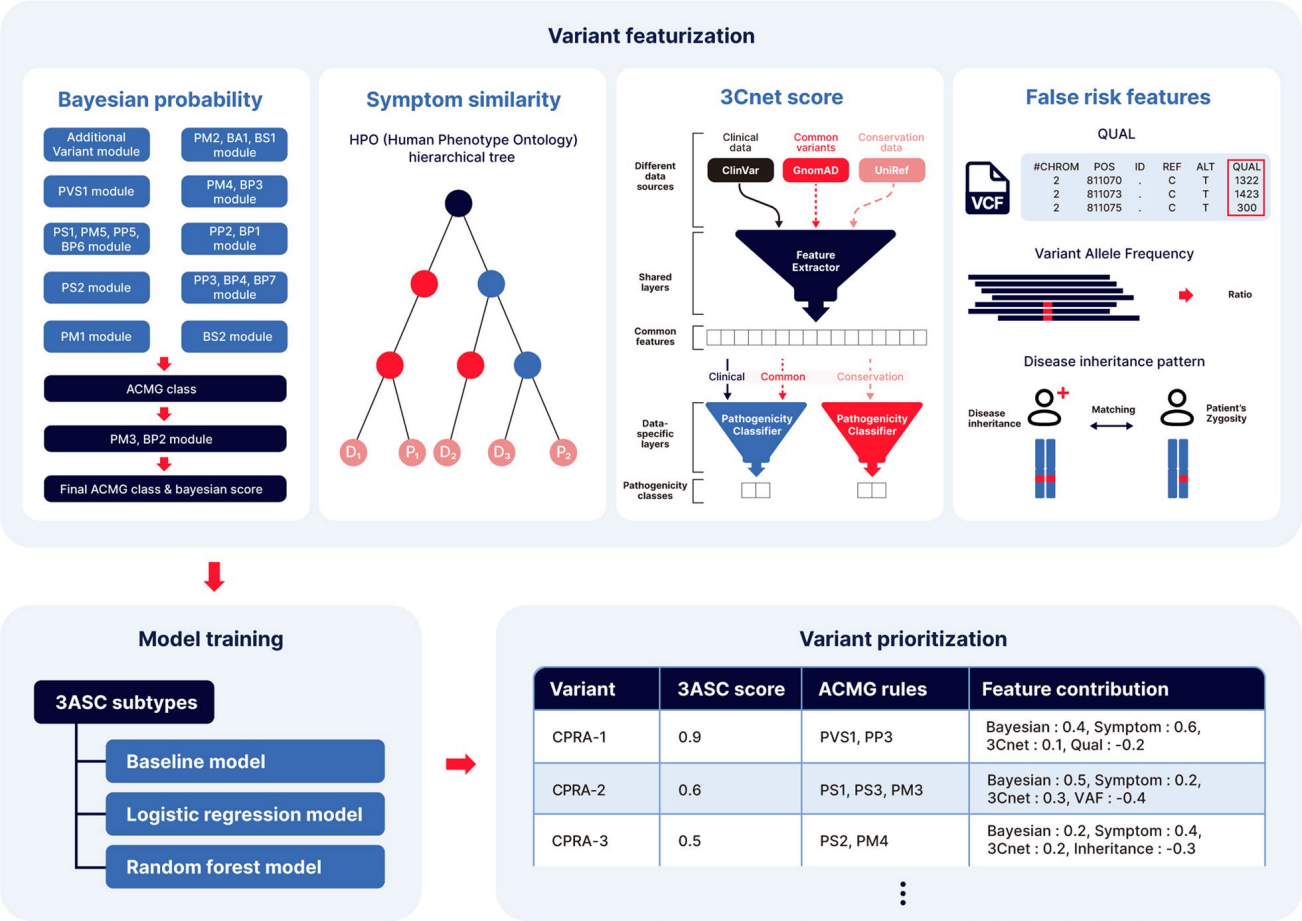
Overview of 3ASC variant prioritization system. The Bayesian score, symptom similarity, the 3Cnet score, and false risk features were trained to build different 3ASC models including the baseline model, logistic regression models, and random forest models. 3ASC prioritizes variants of each patient according to the scores, and annotates ACMG criteria to each variant. The user can interpret the prioritization result based on the ACMG rules and feature contribution for each variant

Here, we report a comprehensive algorithm that prioritizes variants with higher sensitivity than that of previous tools with the added capability of annotating variants with the evidence that was used for prioritization. We integrated four types of features into EVIDENCE [18], an internally developed variant annotation and classification tool. First, the Bayesian score [19] was based on the 28 criteria defined by the ACMG/AMP variant interpretation guidelines. Second, the symptom similarity score [20] quantified the semantic similarity between the known symptoms of a specific disorder and those observed in the patient. The Bayesian score and symptom similarity score were used to prioritize variants based on genotype–phenotype knowledge alongside clinical evidence. Third, the 3Cnet score [21], generated by a trained deep-learning model, provided the likelihood of a given amino acid change impacting the protein function. The 3Cnet scores added extra refinement to the variant classification made by the first two scores. Finally, we adopted features associated with false positives, such as quality control and inheritance patterns, and trained the model to avoid risk using machine learning algorithms. Instead of filtering variants, the model trained with those features could avoid risk of false positives. We named the overall process of variant prioritization 3ASC (Fig. 1).

Additionally, based on the techniques used for explainable AI (X-AI), such as the mean decrease in accuracy (MDA) [22] and Shapley additive explanation (SHAP) [23], 3ASC could explain how each feature contributes to the assessments of genetic variants. The unique traits of 3ASC enable (1) precise variant evaluation based on ACMG guidelines; (2) priority reduction of false positives using a machine learning algorithm; and (3) result explanation based on evidence and feature contribution. 3ASC showed a recall rate of 93.7% among the top 10 variants, which was much more sensitive than Exomiser (81.4%) or LIRICAL (57.1%). Because the system annotates ACMG/AMP criteria for each genetic variant of a patient, clinical geneticists can interpret the result as in the CAGI6 SickKids challenge.

## Results

### Demographic characteristics of in-house patients

The demographic characteristics of the patients are shown in Table 1. Of 5055 patients in our retrospective cohort, 2825 were female (55.9%). In addition, infant patients accounted for the largest proportion in our cohort (n = 1357, 26.8%). Most of their symptoms were in the nervous system (n = 2280, 45.1%), followed by the musculoskeletal system (n = 1722, 34.1%). There were around 122 genetic variants after filtering (see section “Methods”) for each patient on average, resulting in 240,084 unique variants from those patients. For each patient, 4458 patients had one causative variant, while 573 patients had two causative variants and 24 patients had three causative variants. The cases with multiple causative variants include (1) multiple variants were suspected to be causative; (2) causative variants from different alleles for recessive diseases; and (3) causative variants from different genes for digenic diseases.

**Table 1:** Demographic and clinical characteristics of the eligible patients

Variables		Eligible patients (n = 5055)
Gender (n, %)	Male	2230 (44.1)
	Female	2825 (55.9)
Onset age (n, %)	Infancy	1357 (26.8)
	Neonatal	1257 (24.9)
	Childhood	1093 (21.6)
	Adult	888 (17.6)
	Antenatal	269 (5.3)
	Adolescent	183 (3.6)
	Elderly	4 (0.1)
	Unknown	4 (0.1)
Symptoms (n, %)	Nervous system	2280 (45.1)
	Musculoskeletal system	1722 (34.1)
	Head or neck	1231 (24.4)
	Cardiovascular system	1125 (22.3)
	Eye	1057 (20.9)
	Metabolism/homeostasis	847 (16.8)
	Integument	834 (16.5)
	Limbs	719 (14.2)
	Genitourinary system	714 (14.1)
	Growth	794 (15.7)
	Ear	637 (12.6)
	Digestive system	591 (11.7)
	Blood and blood-forming tissues	408 (8.1)
	Endocrine system	384 (7.6)
	Immune system	385 (7.6)
	Respiratory system	208 (4.1)
	Neoplasm	148 (2.9)
	Prenatal development or birth	103 (2.0)
	Constitutional symptom	118 (2.3)
	Breast	35 (0.7)
	Cellular	45 (0.9)
	Voice	25 (0.5)
	Thoracic cavity	1 (0.0)

### Cross validation of ML models compared with the baseline model

**Fig. 2:**
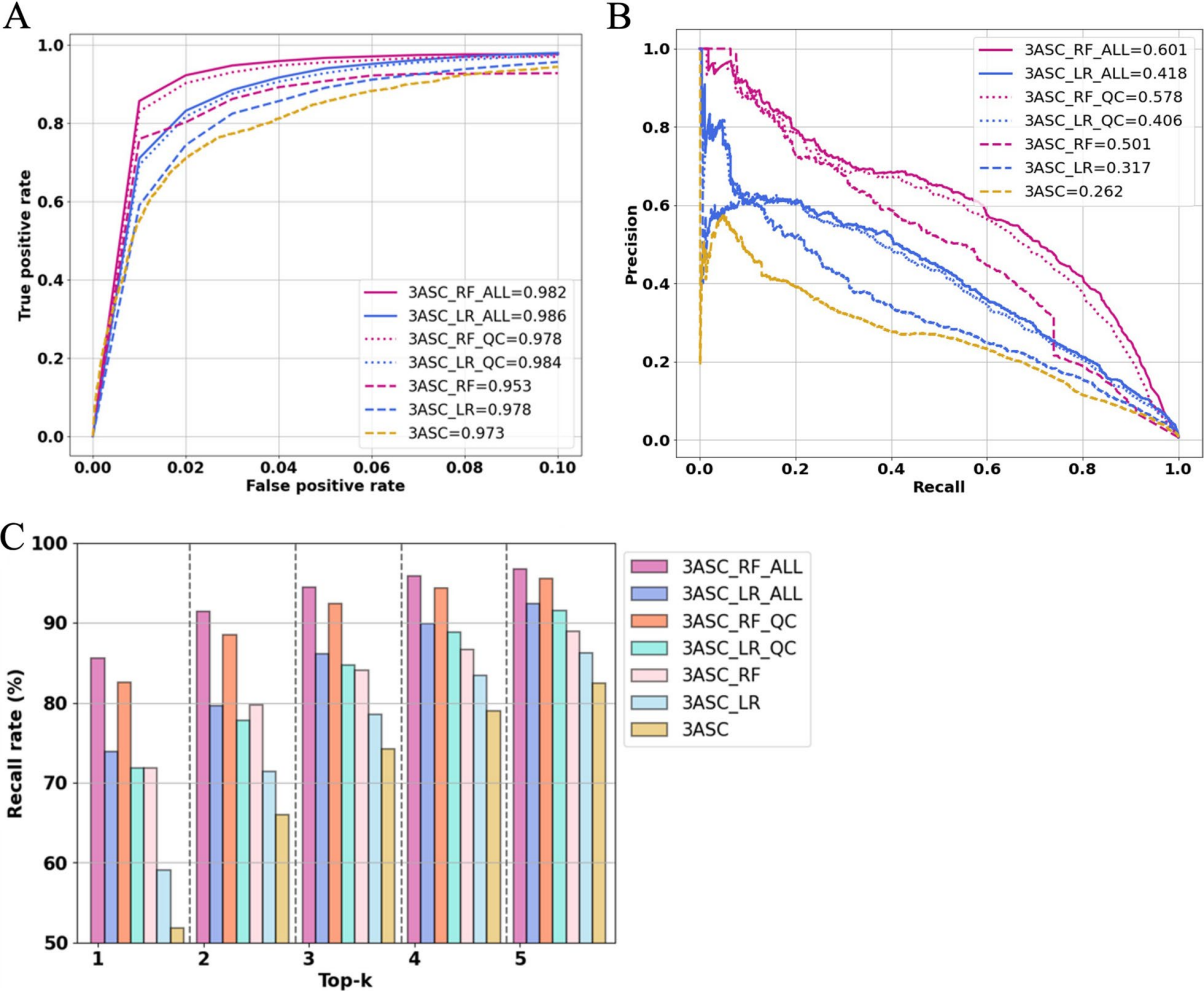
Performance comparison between 3ASC models using cross-validation. **A** Average ROC curve. True positive rates from different folds were averaged for each false positive rate using interpolation. False positive rates ranging from 0 to 0.1 were plotted because the true positive rates were mostly saturated afterwards. **B** Average PR curve. **C** Average top-k recall

Fivefold cross validation of different 3ASC models showed that using all six features improved the prediction performance (Fig. 2). Using machine learning algorithms (3ASC_RF, 3ASC_LR) also improved the performance compared to the baseline model (3ASC). The random forest models were superior for the top 10 variants compared to the other models. However, as most variants were assessed as minimum scores when random forest algorithm was used, approximately 2.5% of causative variants had minimum scores so that they could not be prioritized properly. Nevertheless, the 3ASC_RF_ALL model showed a recall rate of 85.6% for the first variant, which was considerably higher than that of the other models (Table 2). The ROC-AUC of the 3ASC_RF_ALL model showed 98.2%, which was slightly lower than that of the 3ASC_LR_ALL model (98.6%) (Fig. 2A). The result was mainly because of the missed variants which were assessed as minimum scores for the random forest model. On the other hand, 3ASC_RF_ALL showed outperformed the other models by 0.601 of PRAUC (Fig. 2B). In regard to the average number of variants examined before the recall rate reaches 95%, the 3ASC_RF_ALL model could find the causative variants among top 4 variants, followed by top 5 variants for 3ASC_RF_QC and top 7 variants for 3ASC_LR_ALL (Fig. 2C). We selected 3ASC_RF_ALL as the best practice model because a variant prioritization tool is practically used to find the causative variants at the high rank.

**Table 2:** Top-k recall rates for different 3ASC models compared with baseline

Models	Top 1	Top 3	Top 5	Top 10	Top 20	Top 30	Top 100
3ASC (Baseline)	0.518	0.742	0.825	0.911	0.969	0.986	**1.000**
3ASC_LR	0.591	0.785	0.862	0.937	0.977	0.991	**1.000**
3ASC_RF	0.718	0.841	0.889	0.936	0.953	0.960	0.992
3ASC_LR_QC	0.718	0.847	0.915	0.966	0.989	0.995	**1.000**
3ASC_RF_QC	0.825	0.923	0.955	0.976	0.983	0.985	0.998
3ASC_LR_ALL	0.739	0.861	0.924	0.975	**0.993**	**0.997**	**1.000**
3ASC_RF_ALL	**0.856**	**0.944**	**0.967**	**0.981**	0.985	0.988	0.998

The best performance is indicated in bold

### 3ASC models compared with benchmark models by external validation

**Fig. 3:**
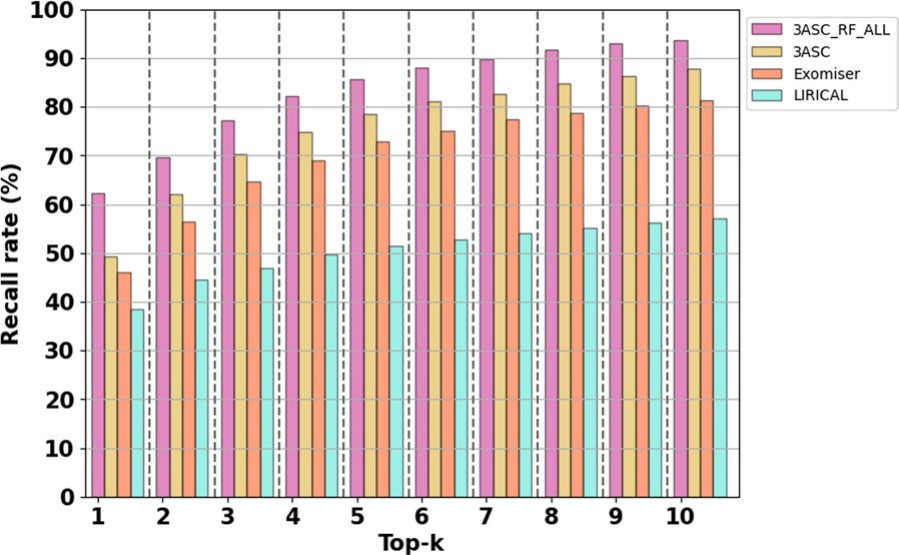
Top-k recall comparison with benchmark models using external validation. The same set of genes and variants were used to compare the performance of variant prioritization without any bias. For Exomiser and LIRICAL, gene scores were first used to prioritize the most probable causal genes and then variants were prioritized using variant scores

Using external validation data, the performance of identifying causative variants in patient genomes was compared between the 3ASC models (Baseline model, best practice model) and the benchmark models (Exomiser, LIRICAL). The results showed that even the baseline model outperformed the benchmark models, even though the model was not trained using patient data. We attempted to reduce the bias in the assessment due to the overfitting when we built the external validation dataset (see section “Methods”). Although no variant in the validation dataset was used to train the 3ASC models, the best practice model (3ASC_RF_ALL) showed a top-5 recall of 85.7% and a top-10 recall of 93.7% (Fig. 3). For the same dataset, Exomiser showed a top-5 recall of 72.9% and a top-10 recall of 81.4%, while LIRICAL showed a top-5 recall of 51.5% and a top-10 recall of 57.1%. Note that the baseline model (3ASC) showed better performance even though no patient data were used to train the model.

### SickKids causal gene prediction result

In the recent CAGI6 SickKids challenge, predictors were asked to prioritize variants based on whole genome sequencing (WGS) data and the phenotype descriptions from children who were referred to The Hospital for Sick Children’s (SickKids) [24–27]. RNAseq-based transcriptome data were also used to assess the impact of variants on gene expression and splicing variation. We participated in the challenge using the baseline model and 3ASC_LR model and were selected as a top-performing team. 3ASC successfully identified causal genes for 10 out of 14 WGS patient cases which were identified by the assessor of the challenge, researchers from SickKids Research Institute (Table 3). Although the 3ASC models did not assess gene expression according to the transcriptome data, they predicted that the causative variants in 6 cases might result in decreased expression based on ACMG criteria PVS1. Among them, two genes (*HDAC8* and *CASK*) had decreased gene expression profiles, which indicates that the rule-based prediction of 3ASC is well aligned with the real-world evidence.

**Table 3:** Results of identification of causative variants for the CAGI6 SickKids challenge

Causal gene	Causal variants found by 3ASC	PVS1 rule applied by 3ASC	Decreased gene expression
HDAC8	**Found**	**Applied**	Found*
CASK	**Found**	**Applied**	Found**
HMGA2	**Found**	**Applied**	Not found
PDHA1	**Found**	**Applied**	Not found
NKX6-2	**Found**	**Applied**	Not found
FOXG1	**Found**	**Applied**	Not found
KCNB1	**Found**	Not applied	Not found
COL12A1	**Found**	Not applied	Not found
EPG5	**Found**	Not applied	Not found
H3F3B	**Found**	Not applied	Not found
ZNF711	Not found	Not applied	Not found
DSG2	Not found	Not applied	Not found
SMAD6	Not found	Not applied	Not found
FBXW7	Not found	Not applied	Not found

The evidence of gene causality is indicated in bold

*log2 transformation for RNAseq read count of the affected sample was compared with the distribution of those of 244 other samples (Z-score = − 10.15)

**log2 transformation for RNAseq read count of the affected sample showed Z-score of − 2.04

### Assessment of feature importance

**Fig. 4:**
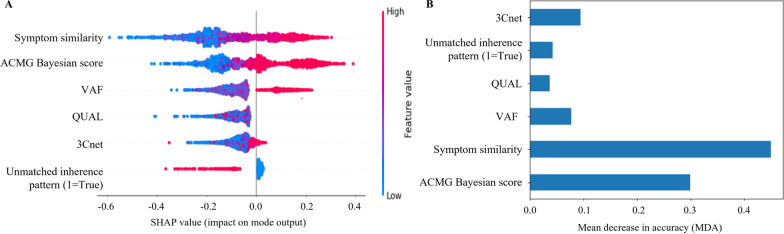
Feature importance measured using SHAP and MDA. **A** SHAP force plot of 3ASC_RF_ALL; **B** Feature importance of 3ASC_RF_ALL

Figure 4A shows SHAP summary plots for the features of the best practice model (3ASC_RF_ALL). Relatively high ACMG Bayesian score, symptom similarity, variant allele frequency (VAF), and 3Cnet score in the dataset showed a positive SHAP value that contributed to the model predicting a high probability for causative variants. In contrast, the variants that did not match the inheritance pattern negatively contributed to the prediction score, which means the feature could reduce the risk of false positives (Fig. 4A). Feature importance was assessed by shuffling each feature while other variables remained constant (MDA). The symptom similarity score caused the greatest decrease in accuracy when shuffled, followed by the ACMG Bayesian score (Fig. 4B). Symptom similarity was the most important feature for the prediction, followed by the Bayesian score and the 3Cnet score. The importance of false risk features, such as variant quality score (QUAL), VAF, and inheritance pattern was relatively low, but still they contributed to the prediction accuracy.

### Case study for a patient with hemophilia A

**Fig. 5:**
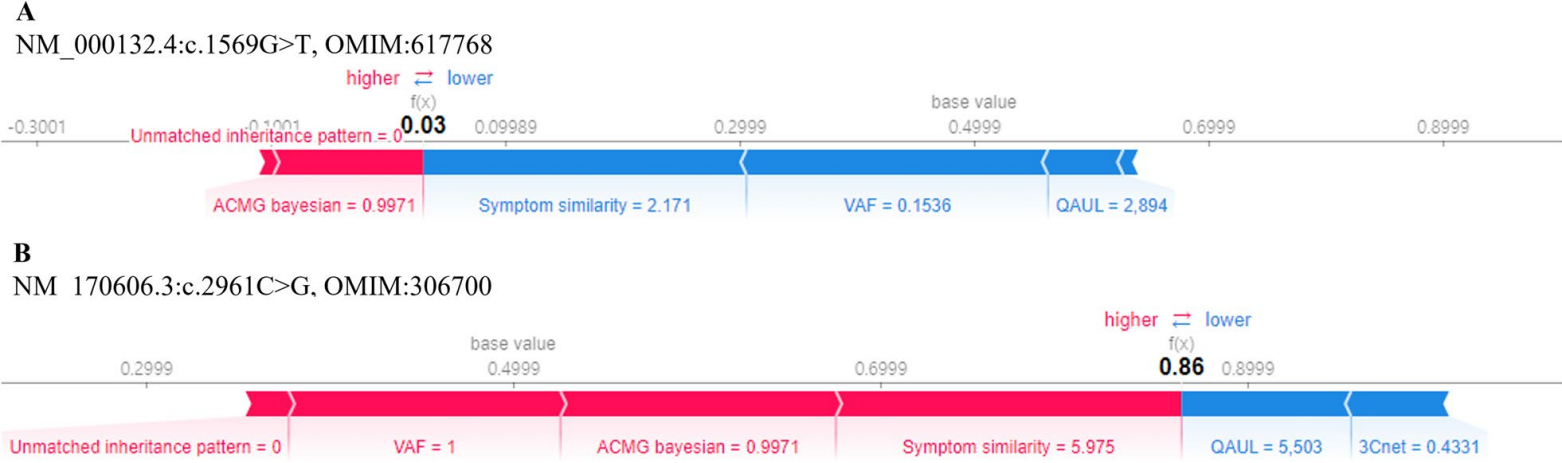
SHAP plot of Individual variants in a patient with hemophilia A. **A** Force plot of a false call variant; **B** Force plot of a confirmative variant

For individual case, a patient with intracranial hemorrhage, hemarthrosis, and Factor VIII deficiency had NM_000132.4:c.1569G > T (ClinVar VCV000439678.18), and was diagnosed with hemophilia A caused by *F8*. Our model also predicted the 3ASC score of the variant (NM_170606.3:c.2961C > G) in *KMT2C*, as 0.03, although the variant was annotated with ACMG Bayesian score of 0.9971 (Fig. 5A). By interpretation with SHAP analysis, the SHAP value of VAF for this variant has a negative contribution to 3ASC score. This is because our model adjusted the score due to the low value of VAF that implies potential false calling. Also, the score was underestimated because of a low symptom similarity score in which patient symptoms differed from another disease.

On the other hand, for the confirmed variant of this patient, our model predicted the 3ASC score as 0.86, which showed high VAF, ACMG Bayesian score, and symptom similarity (Fig. 5B). In detail, we found the high ACMG Bayesian score for pathogenicity of the confirmed variant was induced from the assignment of ACMG rule (PS4, PP3, PM2, PP5, PM4), which is useful information for variant interpretation. For hemophilia A, the matched inheritance pattern showed positive contribution to prediction score because X-linked recessive pattern coincided with zygosity of patient variant.

## Discussion

Some limitations in this study should be improved upon in the future. First, the effectiveness of the 3ASC algorithms was not checked against other database sources, such as Deciphering Developmental Disorders (DDD) [28] or the 1000 Genomes project [29], which are not publicly available. Instead, we prepared an external validation set by dividing in-house patient data according to the time we analysed the samples (before and after 1 September 2022). To address the issue of overfitting, we removed all the variants in the external validation set from the training data. Note that the baseline 3ASC model was not trained by patient data. The priority scores were calculated by multiplication of feature scores with the sigmoid function activation. Additionally, by using the same set of candidate genes and variants, we ensured that the better performance of 3ASC compared to benchmark models was attributed to the superiority of the prioritization algorithms.

3ASC used several features including pathogenicity score derived from several public databases. In particular, pathogenicity scores such as the Bayesian score from the ACMG/AMP guidelines emerged as a strong predictive factor (Fig. 4). To quantify the pathogenicity of a variant, one can use a Bayesian score based on the ACMG/AMP standard guidelines [17]. Another proposed method is a scoring system that calculates pathogenicity as the sum of evidence-based scores [30]. However, our model uses the first method, which is the posterior probability given by the ACMG/AMP-based naive Bayes classifier (Bayesian score). This was the situation when the EVIDENCE annotation tool was developed. At that time, the ACMG/AMP-based scoring system did not exist, so we used the posterior probability and incorporated it into our model for consistency with the annotation tool. In addition, Bayesian systems with scores ranging from 0 to 1 are also well known in research and clinical genetics and have been used in many studies [31–33]. However, for detailed post-hoc analysis in individual prediction, point-based system may be suitable as feature, because additive characteristics of point-based system provide intuitive interpretation for each ACMG/AMP strength of evidence categories.

In addition, 3ASC also used the predictive score from the in-silico prediction tool, 3Cnet, as a feature for variant pathogenicity. In fact, the use of the in-silico prediction tool is already included in the PP3 Rule within the ACMG/AMP guidelines, but we used it as a feature again. The strength of evidence of the PP3 rule may be underestimated because it is at the supporting level, so the ACMG Bayesian has a low value. Therefore, as the prediction can be suboptimized with the Bayesian score partially derived from PP3, we used in-silico prediction directly at the feature level. There are studies suggesting that in-silico prediction is more robust than expected, suggesting a higher strength [34, 35]. We identified the prioritization score was improved when 3ASC used in-silico predictive score by conducting ablation tests without the 3Cnet score (Additional file 1: Supplementary document 1).

Although the random forest models of 3ASC showed the best sensitivity for high-ranking variants, a number of variants were scored as zero (approximately 90%). Some of the causative variants (approximately 2.5%) were also neglected, which resulted in a failure to find all positives even after top 100 variants. This is because of the class imbalance between positive and negative variants. Among hundreds of candidate variants in the genome of each patient, only 1–3 variants cause the genetic disorders, which makes the random forests underestimate the pathogenicity of candidate variants. Therefore, the random forest models need to be adjusted to address the class imbalance. One way to do so would be combining the logistic regression models to prioritize variants in lower ranks.

The 3ASC models we presented in this study focus on finding causative variants, but they are not optimized to determine whether each patient has a Mendelian disorder. The score distribution of causative variants could vary depending on the symptoms and the candidate diseases of the patients. Therefore, there are certain risks determining the pathogenicity based solely on the scores of this model. For the practical use, we recommend that clinical geneticists manually examine each variant prioritized by this method with the annotated ACMG criteria and feature contribution of the model. In this study, we chose conventional machine learning algorithms such as logistic regression and random forests. This is mainly because the features for variants can be obtained in the form of uniform tabular data. For future works utilizing unstructured data regarding variant interpretation, such as the context from the literature, deep neural networks might offer better performance and feature flexibility.

## Conclusions

3ASC is an automated pipeline for variant prioritization that follows the ACMG/AMP guidelines for variant interpretation. It annotates the ACMG criteria based on evidence from various databases with different strengths of evidence ranging from supporting evidence to very strong evidence. The Bayesian score is then calculated using the annotation, which represents the posterior probability score for the pathogenicity of each variant. Unlike other variant prioritization methods, which mainly utilize in-silico prediction for the pathogenicity score, this approach could not only increase the sensitivity of the prioritization but also enable precise interpretation of the variant pathogenicity. In addition, we integrated various features related to false-positive variants, such as quality control and inheritance patterns, to train machine learning algorithms. The techniques of explainable AI were applied to the models so that we could examine why each variant had such a high/low priority based on the feature contribution.

## Methods

### Patient data preparation

In this study, exome sequencing variant data generated from 5055 patients at a single reference laboratory in South Korea were used. These patients were referred from ~ 50 countries between March 16, 2021 and February 10, 2023 because they were suspected to have rare genetic disorders and were found with pathogenic or likely pathogenic diagnostic variants. Patient samples were received as EDTA blood, buccal swabs or extracted genomic DNA. All protein coding regions of known human genes (~ 22,000) were captured by xGen Exome Research Panel v2 (Integrated DNA Technologies, Coralville, Iowa, USA) and sequenced with Novaseq6000 (Illumina, San Diego, CA, USA) as 150 bp paired-end reads. Binary base call (BCL) files generated from sequencing were converted and demultiplexed to generate FASTQ files. The sequencing reads in the FASTQ files were aligned to the human reference genome (GRCh37/hg19 from NCBI, February 2009) using BWA-MEM (v.0.7.17) [36]. Aligned BAM files were sorted and extracted using the statistical metrics by samtools (v.1.9) [37], Variant call format (VCF) files were generated from BAM files following the GATK best practices (GATK v3.8) [38]. Variants were then annotated with Ensembl Variant Effect Predictor (VEP v104) [39] and classified according to the ACMG/AMP guidelines using 3billion’s bioinformatics pipeline EVIDENCE as previously described [40]. For each patient, variants meeting the following conditions were filtered out: (1) variants with the allele frequency > 5% of in gnomAD 2.0 [41]; (2) likely benign/benign variants as classified by the ACMG guidelines; (3) variants in genes that are not associated with a monogenic disorder in Online Mendelian Inheritance in Man (OMIM) [42] Human Phenotype Ontology (HPO), OrphaNet [43], Clinical Genomic Database (CGD) [46]. Finally, a total of 4,840 variants deemed disease-causing were submitted to ClinVar [44]. Phenotypes and variant information of the selected 5055 patients analysed throughout the manuscript are presented as in-house data.

### Evidence system: ACMG Bayesian score

EVIDENCE, the bioinformatics software pipeline, classifies variant pathogenicity with schema based on the ACMG guidelines incorporating daily automatically updated databases, including public databases, in-house variant databases and manually curated literature databases [18]. The variant classification schema assigns each variant to one of five classification tiers, including benign (B), likely benign (LB), pathogenic (P), likely pathogenic (LP) and variant of uncertain significance (VUS). It combines and weighs the strength of the evidence, which is divided into stand-alone (A), very strong (VS), strong (S), moderate (M) and supporting (P) [47, 48]. To exclude variants that are obviously not the disease-causing mutations, variants with BA1 evidence whose alleles are abundant in the population, and variants classified as benign/likely benign were removed from further analysis.

Tavtigian et al. have proposed a Bayesian statistical framework for the ACMG guideline-based variant interpretations to quantitatively explain the five-tier variant pathogenicity classification [19]. To create the variant pathogenicity feature assigned following ACMG guideline, we adjusted the Bayesian statistical framework to mutation and calculated the posterior probability score for the pathogenicity of each variant according to the strength of the evidence. Given the prior probability of the variant pathogenicity ($Prior\_P$) and odds of pathogenicity ($OddsPath$) for very strong evidence (${O}_{PVS}$), if N was the number of criteria with a given strength of evidence category, the posterior probability of the variant pathogenicity ($Post\_P$) was determined as defined below (Eq. 1).

1 $$\begin{gathered} OddsPath = O_{PVS}^{{\left( {\frac{NPP}{8} + \frac{NPM}{4} + \frac{NPS}{2} + \frac{NPVS}{1} - \frac{NBP}{8} - \frac{NBS}{2}} \right)}} \hfill \\ Post\_P = \frac{OddsPath*Prior\_P}{{\left( {\left( {OddsPath - 1} \right)*Prior\_P + 1} \right)}} \hfill \\ \end{gathered}$$

The prior probability of the variant pathogenicity and odds of pathogenicity for very strong evidence were given to be 0.1 and 350 respectively, as suggested by Tavtigian et al. [19] The calculated posterior probability score for the pathogenicity of each variant is used for one of the following 3ASC features, called the ACMG Bayesian score.

### Symptom similarity: gene-similarity upweighted Resnik similarity

Patients with RDs usually assume that one or two genes are dysfunctional. If this gene is dysfunctional, the functions that this gene is responsible for will be consistently malfunctioned. For this reason, symptom similarity between a patients’ symptoms and those of the disease has been widely used to determine the causal genes. For this reason, we assumed that symptom similarity could be improved when jointly considered between patient phenotypes and phenotypes in gene. For example, patients with dysfunction of the *F10* gene encoding the vitamin K-dependent coagulation factor X have more phenotypes related to coagulation than patients with dysfunction of haematopoietic-related genes, or hepatic function-related genes (e.g. symptoms of hepatocellular carcinoma induced by the *APC* gene or leukemia by *ABL1*).

We calculated symptom similarity using gene-similarity upweighted Resnik similarity, which is a modified form of two-sided Resnik similarity that additionally considers the relevance between the patient’s reported phenotype and each candidate disease’s known causal gene. Phenotypes are represented using HPO terms (version 2023-01-27), diseases using the union set of monogenic Online Mendelian Inheritance in Man (OMIM) and Orphanet terms, and genes are represented as defined by the Entrez database [49]. Given an HPO phenotype $p$, a set of phenotypes $Q$, a disease-to-phenotype mapping $D$ for the case of disease $k$, and a gene-to-disease mapping $G$ for the case of gene $m$ are defined as shown below.

2 $$\begin{aligned} Q & = \left\{ {p_{q,1 } , \ldots ,p_{q,i } } \right\} \\ D_{k} & = \left\{ {p_{k,1} , \ldots ,p_{k,j } } \right\} \\ G_{m} & = \{ D_{m,1,} , \ldots ,D_{m,k } \} \\ \end{aligned}$$

The gene-upweighted Resnik similarity function Sim_upweighted_ is the product of the symptom-gene similarity function Sim_gene_ and the two-sided Resnik similarity function Sim_two-sieded resnik_ and, such that

3 $$Sim_{upweighted } \left( {Q,D_{k } } \right) = \;Sim_{gene} \left( {Q,G_{m } } \right) * Sim_{two - sided resnik} \left( {Q, D_{k} } \right), where D_{k } \in G_{m }$$

and

4 $$Sim_{gene } \left( {Q, G_{m } } \right) = 1 + \frac{{e^{{ Sim_{sym\_resnik } \left( {Q,G_{m } } \right)}} }}{{\mathop \sum \nolimits_{m}^{M} e^{{Sim_{sym\_resnik } \left( {Q,G_{m } } \right)}} }}$$

The set-level semantic similarity between two sets of phenotypes$Q=\{{p}_{q,1},\dots ,{p}_{q,i }\}$,$D=\{{p}_{k,1 }, \dots ,{p}_{k,j } \}$

5 $$Sim_{{two{ - }sided resnik}} \left( {Q,D} \right) = \frac{1}{2}Sim_{{one{ - }sided}} \left( {Q \to D} \right) + \frac{1}{2}Sim_{{one{ - }sided }} \left( {D \to Q} \right)$$

The one-sided semantic similarity is defined as:

6 $$Sim_{{one{ - }sided}} \left( {Q \to D} \right) = avg\left[ {\mathop \sum \limits_{t \in Q} \max_{{t_{2} \in D}} sim\left( {t_{1 ,} t_{2 } } \right)} \right]$$

We used the term-level semantic similarity defined by Resnik [50].

### 3Cnet: variant pathogenicity predicted by a deep neural network

A deep neural network trained with evolutionary constraints, 3Cnet [21], was used to predict variant pathogenicity and add extra refinement to variant prioritization. The 3Cnet score indicates the probability of variants being pathogenic according to the amino acid change in the context of the protein sequence. There are three different sources of data representing the pathogenicity of the variants, which we named as clinical, common, and conservation data. The clinical data indicate the known pathogenic or benign variants reported in the ClinVar database. Common variants are variants with high allele frequency, considered as benign variants. Conservation data are the artificial variants generated based on evolutionary constraints imposed upon each gene. The 3Cnet network was trained using those pathogenicity data based on multitask learning so that overfitting of the network could be avoided. Variants must be represented in terms of HGVSp annotation [51] to be evaluated by 3Cnet. In this study, we utilized the VEP annotator to obtain the HGVSp annotation for each variant. For those variants that cannot be annotated to HGVSp or evaluated using 3Cnet, we imposed the average score (~ 0.206) of the 216,960 variants from in-house patient data. The types of variants that can be evaluated by the 3Cnet include missense, deletion, insertion, INDEL, duplication, extension (both 5 prime and 3 prime), frameshift, stop gain, start loss, and synonymous variants. Codes for 3Cnet are freely available to noncommercial users (https://github.com/KyoungYeulLee/3Cnet/).

### QC-related features: variant calling quality factors

We added the features related to variant calling quality from VCFs (QC-related features). Although 3ASC can predict a highly pathogenic variant, it would be a false positive to confirm an artefactual variant as a disease-causing variant. Based on this motivation, we modeled the machine learning system to adjust the risk by adding Variant Allele Frequency (VAF) and the variant quality score (QUAL) in VCF as features. VAF is the percentage of sequence reads observed to match specific DNA variants divided by overall coverage at that locus [52]. Low VAF value may be potential errors due to incorrect base calls or alignment. In addition, the variant quality score (QUAL) is generated during variant calling. For example, QUAL = 20 means there is 1% of probability that there is no variant at the site, and QUAL = 50 indicates 0.00001 of probability.

### Disease inheritance pattern: incomplete zygosity

In addition to the pathogenicity of variants and disease similarity, the disease inheritance pattern of genetic variants for RDs was elucidated. For clinical reporting, variant interpretation to identify disease-causing variants includes the match between the disease inheritance pattern, and the zygosity of the allele [53]. We implemented this by comparing the zygosity of patient variants with inheritance patterns from OMIM, OrphaNet, and CGD. We treated this feature as a Boolean value, true for unmatched inheritance pattern between disease and zygosity of the patient variant; false for matched inheritance pattern between them.

### 3ASC models

We defined various models to prioritize genetic variants based on the features related to the clinical interpretation of variants. We started with the baseline model, which was a simple combination of the Bayesian score, symptom similarity, and the 3Cnet score without training patient data to optimize the model. By doing so, we could guarantee that the model was not overfitted to the in-house patient data. The baseline model used simple multiplication of the scores with sigmoid activation for symptom similarity and the 3Cnet score. For robust prediction from variant with dissimilar disease which has low value of symptom similarity, we heuristically chose the subtraction value of 2.0 from the distribution of symptom scores. Additionally, 3Cnet scores were also transformed by a sigmoid function to avoid degradation of false-negative variants that have 3Cnet scores of zero but still have the possibility of causing diseases. Hundreds of variants from each patient were scored by the final 3ASC scores and prioritized accordingly (Eq. 7). As the Bayesian score and symptom similarity score would be different depending on the candidate disease for each variant, the maximum 3ASC score was selected to measure variant-level prediction performance.

7 $$\begin{aligned} & \sigma \left( x \right) = \;1 / \left( {1 + exp\left( { - x} \right)} \right) \\ & 3ASC \,score = Bayesian \,score \times \sigma \left( {Symptom \,similarity - 2} \right) \times \sigma \left( {3Cnet \,score} \right) \\ \end{aligned}$$

To further optimize the model so that it could prioritize the variants with higher sensitivity, we trained machine learning models using in-house patient data. First, we trained logistic regression models with the Bayesian score, symptom similarity, and the 3Cnet score as input features. Based on the LogisticRegression classifier defined in the scikit-learn python package, we set the class_weight option as “balanced” and the max_iter option as 500. We also trained random forest models using the same features. RandomForestClassifier from the scikit-learn package was used with the n_estimators option set as 500 and the class_weight option set as “balanced”. Other options for those models remained as default.

QC-related features and inheritance pattern features were also included for other machine learning models (Table 4). We validated and compared the performance of each model using fivefold cross-validation. As a result, a random forest model trained with the Bayesian score, symptom similarity, the 3Cnet score, QC-related features, and inheritance pattern features showed the best performance. We defined these models as the best practice model (3ASC_RF_ALL). Finally, the performance of the baseline model and the best practice model was compared with that of benchmark models using external validation.

**Table 4:** Various 3ASC models with different algorithms and features

Model name	Algorithms	Data training	Bayesian score	Symptom similarity	3Cnet score	QC-related features	Inheritance pattern
3ASC (Baseline)	Multiplication	No	Yes	Yes	Yes	No	No
3ASC_LR	Logistic regression	Yes	Yes	Yes	Yes	No	No
3ASC_LR_QC	Logistic regression	Yes	Yes	Yes	Yes	Yes	No
3ASC_LR_ALL	Logistic regression	Yes	Yes	Yes	Yes	Yes	Yes
3ASC_RF	Random forest	Yes	Yes	Yes	Yes	No	No
3ASC_RF_QC	Random forest	Yes	Yes	Yes	Yes	Yes	No
3ASC_RF_ALL	Random forest	Yes	Yes	Yes	Yes	Yes	Yes

### Benchmark models

Based on the benchmark tests and code availability mentioned from the previous literature [54], we selected Exomiser and LIRICAL as benchmark models to compare the variant prioritization performance. Exomiser version 13.0.1 with data version 2109 was executed using Java version 18.0.1.1. VCF file paths and HPO values were substituted at runtime. Further details regarding its configuration can be found in Additional file 2: Supplementary document 2. LIRICAL 1.3.4 was also executed using Java 18.0.1.1. VCF file paths and HPO values were substituted at runtime. We used hg19 for the genomeAssembly and Exomiser database version 2109_hg19. To prevent bias caused by data preprocessing, the same set of genes and variants parsed and filtered from the VCF files was used for all the models. Both Exomiser and LIRICAL calculate gene scores and variant scores separately. For example, Exomiser generates a gene score file and a variant score file for each VCF file. The final combined score is given in the gene score file at the column named “EXOMISER_GENE_COMBINED_SCORE”. The variant score of the Exomiser is given in the variant score file at the column named “EXOMISER_VARIANT_SCORE”. On the other hand, LIRICAL generates a single result file for each VCF file with genes prioritized by the posttest probability given at the column named “posttestprob”. At the column named “variants”, the pathogenicity score for each variant is given. As those scores would be different according to the candidate diseases, the maximum scores were used for each gene and variant. We first prioritized genes based on gene scores and then prioritized variants for each gene using variant scores. By doing so, we ensured that the prioritization performance of the 3ASC models and benchmarks models could be compared based on the same number of candidate genes and variants.

### Model comparison and interpretation

Top-k recall, a common metric for recommendation systems, was used to evaluate model performance. Top-k recall refers to the proportion of hits, where a hit indicates the predicted rank of the causative variant among other variants of the patient is within a predefined cutoff k. For example, if a model found one causative variant within rank 5 among 2 causative variants of the patient, the top-5 recall for the patient become 0.5 (1 hit and 1 miss). Some patients with dual diagnosis may have multiple confirmed causative variants, which lead to the number of hits for a patient may exceed the cutoff k. In such cases, the recall rate cannot reach 1 even if the prioritization is perfect, because the ranks of a few causative variants always exceed the cutoff. To address the issue, we revised the denominator of the recall as the minimum value between the number of causative variants or cut-off k for each patient. Then, Top-k recall is averaged across patients for the same cutoff k.

To evaluate the consistency in the model’s performance, fivefold cross-validation (training set: 80%, test set: 20%) was performed by randomly dividing patients into fivefold. Firstly, before constructing the fivefold dataset, we split the specific date (2022-09-01) on which the sample was analysed, and we further excluded the variants shared by both training and test samples in the training dataset to avoid overfitting of the model. We used 4,141 of patient’s data as 5 folds for cross validation, and 914 of patient’s data as external validation dataset. The performance of the 3ASC models, including the baseline model and the best practice models, was then compared with that of Exomiser and LIRICAL based on top-k recall rates. In addition, despite cross-validation, the same type of causal gene may be the training data and test data, potentially leading to performance gains due to data leakage. We constructed 5 folds based on genes, separating patients with no overlapping genes between each fold (Additional file 3: Supplementary document 3).

### Calculation of feature contribution

For reliable AI, we conducted post hoc analysis with model-agnostic X-AI (eXplainable AI) techniques, including SHAP (Sapley additive explanations) and permutation feature importance. SHAP provides an importance value (SHAP value) of each feature for a particular prediction by estimating the conditional expectation of features [55]. In addition, permutation feature importance was measured by the mean decrease in accuracy (MDA), which refers to the magnitude of the average decrease in accuracy by shuffling values in a column.

### Supplementary Information

**Additional file 1**. Ablation test between 3ASC models using 3Cnet score as feature and those without the score.

**Additional file 2**. The detail setting of Exomiser when it was used as the benchmark model to compare the performance of causal gene discovery.

**Additional file 3**. Cross-validation between different disease-causing genes compared with that between different patients.

## Data Availability

The variant interpretation data of in-house patients are available from ClinVar with the submitter 3billion (https://www.ncbi.nlm.nih.gov/clinvar/submitters/507830). All datasets and models used and/or analysed during the current study are available from the corresponding author on reasonable request.

## Abbreviations

RDs: Rare disorders
HPO: Human phenotype ontology
VCF: Variant call format
LR: Likelihood ratio
ACMG/AMP: The American College of Medical Genetics and Genomics and the Association for Molecular Pathology
X-AI: Explainable AI
MDA: Mean decrease in accuracy
SHAP: Shapley additive explanation
CAGI: Critical assessment of genome interpretation
VAF: Variant allele frequency
QUAL: Variant quality score
OMIM: Online Mendelian inheritance in man
CGD: Clinical genomic database
