# Supplementary material for: Explicable prioritization of genetic variants by integration of rule-based and machine learning algorithms for diagnosis of rare Mendelian disorders
Source: Hum Genomics. 2024 Mar 21;18:28. doi: 10.1186/s40246-024-00595-8 (PMC10956189; doi:10.1186/s40246-024-00595-8)
Supplement: Supplementary file 1 — Additional file 1. Ablation test between 3ASC models using 3Cnet score as feature and those without the score. [file 40246_2024_595_MOESM1_ESM.docx]

**Supplementary document 1**

We performed an ablation test to identify the performance improvement of variant prioritization depending on whether or not 3Cnet scores are used as features in the model. In this ablation test, we found that regardless of the type of model (logistic regression or random forest), using 3Cnet scores in feature level improved performance (Supplementary figure 1).


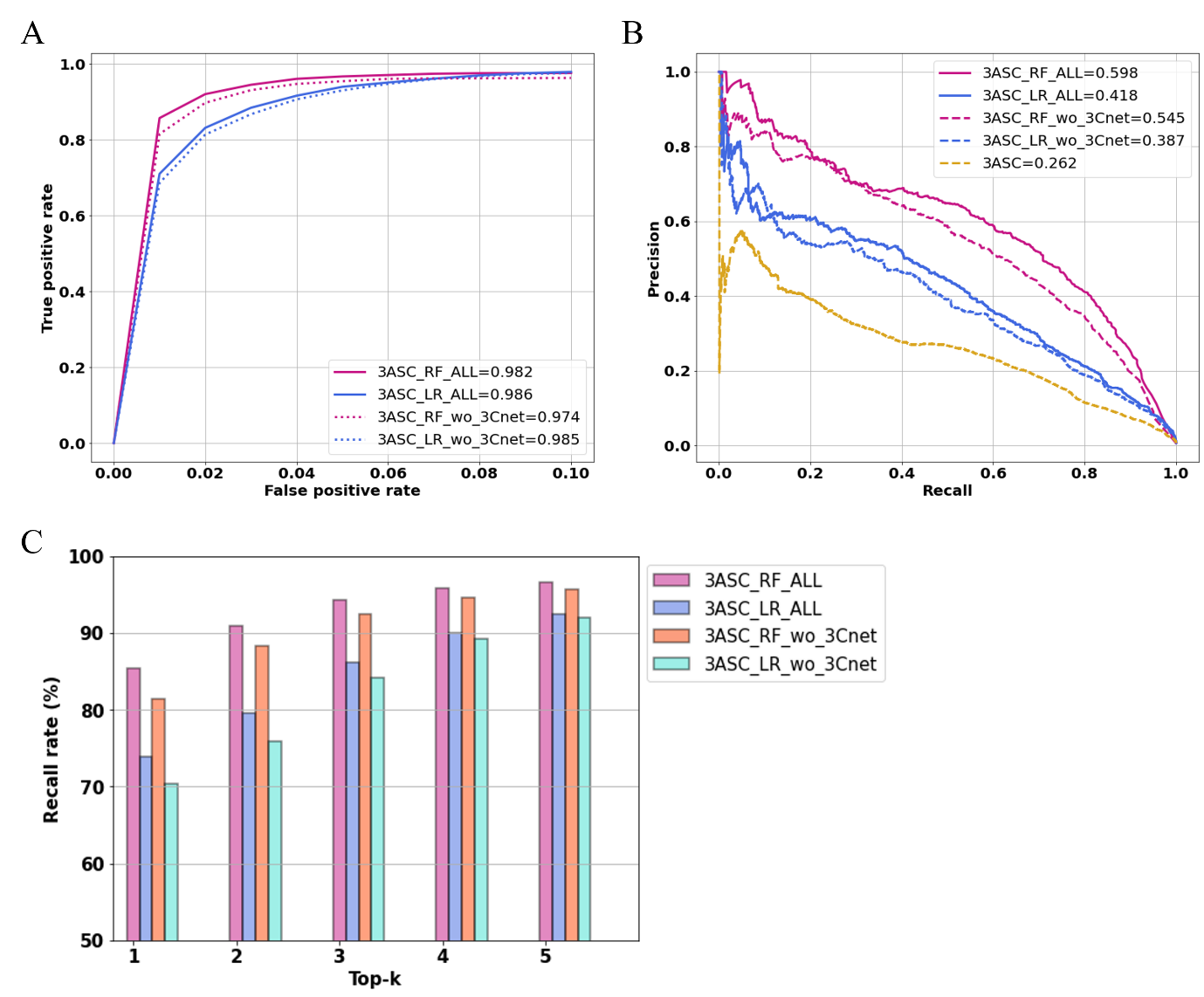


Supplementary figure 1. Performance comparison between 3ASC models with and without 3Cent score as feature. 3ASC models with 3Cnet regardless model type such as LR, or RF outperform those without score for call metrics. A) PR curve. B) ROC curve. C. Top-k recall
