# Supplementary material for: Explicable prioritization of genetic variants by integration of rule-based and machine learning algorithms for diagnosis of rare Mendelian disorders
Source: Hum Genomics. 2024 Mar 21;18:28. doi: 10.1186/s40246-024-00595-8 (PMC10956189; doi:10.1186/s40246-024-00595-8)
Supplement: Supplementary file 2 — Additional file 2. The detail setting of Exomiser when it was used as the benchmark model to compare the performance of causal gene discovery. [file 40246_2024_595_MOESM2_ESM.docx]

**Supplementary document 2**

Exomiser 13.0.1

- hg19 (version 2109)
- analysisMode: PASS_ONLY
- pathogenicitySources: "POLYPHEN, MUTATION_TASTER, SIFT"
- variantEffectFilter: {
- remove: [
- FIVE_PRIME_UTR_EXON_VARIANT,
- FIVE_PRIME_UTR_INTRON_VARIANT,
- THREE_PRIME_UTR_EXON_VARIANT,
- THREE_PRIME_UTR_INTRON_VARIANT,
- NON_CODING_TRANSCRIPT_EXON_VARIANT,
- NON_CODING_TRANSCRIPT_INTRON_VARIANT,
- CODING_TRANSCRIPT_INTRON_VARIANT,
- UPSTREAM_GENE_VARIANT,
- DOWNSTREAM_GENE_VARIANT,
- INTERGENIC_VARIANT,
- REGULATORY_REGION_VARIANT
- ]
- }
- frequencyFilter: {maxFrequency: 2.0}
- pathogenicityFilter: {keepNonPathogenic: true}

Java 18.0.1.1

- Initial heap size 2GB, max heap size 4GB
