# Supplementary material for: Explicable prioritization of genetic variants by integration of rule-based and machine learning algorithms for diagnosis of rare Mendelian disorders
Source: Hum Genomics. 2024 Mar 21;18:28. doi: 10.1186/s40246-024-00595-8 (PMC10956189; doi:10.1186/s40246-024-00595-8)
Supplement: Supplementary file 3 — Additional file 3. Cross-validation between different disease-causing genes compared with that between different patients. [file 40246_2024_595_MOESM3_ESM.docx]

**Supplementary document 3**

We additionally conduct cross-validation based on genes. Because a single rare disease can affect multiple systems, it is hard to assign a disease to a single strain for cross-validation. Instead, we constructed 5 folds based on disease-causing genes, which has the confirmed variant, with no overlapping genes between each fold. We collected unique 1,300 of disease-causing genes from 4,141 of patients in 5 folds, and divided patients according to genes in each fold. We identified less than 0.01 of decreased AUROC compared to patient level cross-validation (Supplementary figure 2-A). Also, the model showed decreased PRAUC up to 0.064. However, top-k recall referring prioritization performance also showed less than 0.01 of top k recall compared to patient level cross validation for all model (Supplementary figure 2).


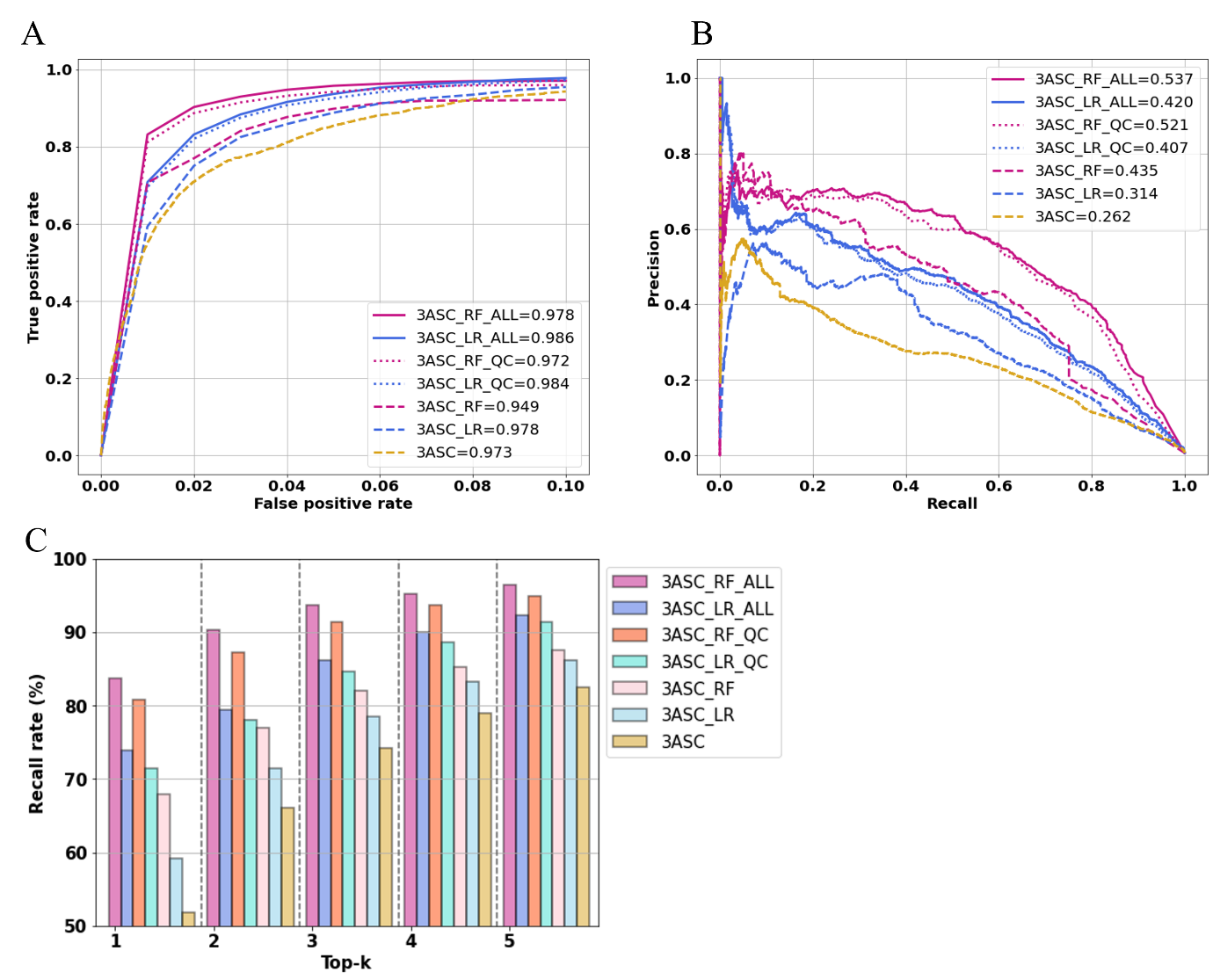


Supplementary figure 2. Gene level cross-validation performance. A) average AUROC. B) average PRAUC. C) average Top-k recall
